# The linkage between inflammation and fibrosis in muscular dystrophies: The axis autotaxin–lysophosphatidic acid as a new therapeutic target?

**DOI:** 10.1007/s12079-021-00610-w

**Published:** 2021-03-10

**Authors:** Felipe S. Gallardo, Adriana Córdova-Casanova, Enrique Brandan

**Affiliations:** 1 Centro de Envejecimiento y Regeneración, CARE Chile UC, Santiago, Chile; 2 Departamento de Biología Celular y Molecular, Facultad de Ciencias Biológicas, Pontificia Universidad Católica de Chile, Santiago, Chile; 3 Fundación Ciencia & Vida, Santiago, Chile

**Keywords:** Muscular dystrophies, Fibrosis, Inflammation, Lysophosphatidic acid, Autotaxin

## Abstract

Muscular dystrophies (MDs) are a diverse group of severe disorders characterized by increased skeletal muscle feebleness. In many cases, respiratory and cardiac muscles are also compromised. Skeletal muscle inflammation and fibrosis are hallmarks of several skeletal muscle diseases, including MDs. Until now, several keys signaling pathways and factors that regulate inflammation and fibrosis have been identified. However, no curative treatments are available. Therefore, it is necessary to find new therapeutic targets to fight these diseases and improve muscle performance. Lysophosphatidic acid (LPA) is an active glycerophospholipid mainly synthesized by the secreted enzyme autotaxin (ATX), which activates six different G protein-coupled receptors named LPA_1_ to LPA_6_ (LPARs). In conjunction, they are part of the ATX/LPA/LPARs axis, involved in the inflammatory and fibrotic response in several organs-tissues. This review recapitulates the most relevant aspects of inflammation and fibrosis in MDs. It analyzes experimental evidence of the effects of the ATX/LPA/LPARs axis on inflammatory and fibrotic responses. Finally, we speculate about its potential role as a new therapeutic pharmacological target to treat these diseases.

## Introduction

Under conditions of damage and healing, the skeletal muscle presents a formidable capability to regenerate. An acute inflammatory reaction and the deposition of extracellular matrix (ECM) occur as scaffolding responses to form new muscle fibers. Under chronic damage conditions, the muscle fibers are partially replaced by new ones, and persistent inflammation ensues. These events are followed by overexpression of pro-fibrotic factors, transformation or activation of specialized resident ECM-producing cells, and exacerbated ECM accumulation. These characteristics define fibrosis, a hallmark of several pathological conditions such as skeletal muscle dystrophies, motor-neuron diseases, and models of denervation and myotrauma.

There have been several attempts to reduce inflammation and fibrosis in different animal models, which have been revised in excellent comprehensive reviews (Mahdy 2019; Mann et al. 2011; Smith and Barton 2018; Dort et al. 2019; Tidball 2011). Inflammation and fibrosis are common characteristics of several chronic diseases that develop in lung, liver, kidney, heart, and other organs (Wynn and Vannella 2016; Wynn 2011).

In the last few years, there has been increased interest in lysophosphatidic acid (LPA), an active glycerophospholipid signaling lipid; their signaling receptors (LPA_1_ to LPA_6_); and autotaxin (ATX), one of the enzymes responsible for its synthesis. This ATX/LPA/LPARs axis has been involved in inflammatory and fibrotic responses in several organs and tissues and as a result of these investigations, some molecules with pharmacological potential have been identified.

This paper reviews the characteristics of inflammation and fibrosis in skeletal muscle dystrophies and the role of the ATX/LPA/LPARs axis in these two responses. We also speculate about the therapeutic value of agonists/inhibitors of this axis for their potential use in muscular dystrophies.

## Muscular dystrophies

Muscular dystrophies (MDs) are a heterogeneous group of inherited skeletal muscle diseases primarily characterized by progressive muscle weakness and degeneration, leading to reduced lifespan of the affected individuals. The most common and severe is Duchenne muscular dystrophy (DMD), an X-linked recessive type of MD (Hoffman et al. 1987). DMD affects 1 in 3500 live-born males and causes severe loss of muscle strength and learning disabilities, potentially leading to death due to respiratory or cardiac failure. The common characteristics of MDs are persistent inflammation, fibrosis (excessive accumulation of ECM), and reduced tissue regeneration capability. Histologically, degenerating muscle fibers are replaced with fibrofatty tissue (Mann et al. 2011), affecting muscle architecture and functionality.

DMD is caused by mutations in the 2.5 Mb *DMD* (loci) gene, which encodes a 427 kDa membrane-associated cytoplasmic protein called dystrophin that is essential for skeletal muscle cell-ECM linkage. For this reason, dystrophin is constantly expressed by differentiated myotubes and myofibers. Surprisingly, the skeletal muscle stem cells responsible for muscle regeneration also express dystrophin, which has a distinct role in this cell type (discussed below) (Dumont et al. 2015). Some mutations in the *DMD* sequence result in reading-frame shifting, causing the translation of a truncated protein and, consequently, loss of expression. However, many DMD mutations correlate with the severity of disease onset (Aartsma-Rus et al. 2006). Indeed, Becker MD, the DMD-linked mild-form of MD, is characterized by a mutation in the *DMD* gene which, unlike DMD, the skeletal muscle is able to express a truncated form of dystrophin.

Dystrophin is a member of the dystrophin-associated glycoprotein complex (DGC), formed by proteins of the sarcoglycan (α-, β-, γ- and δ-SG) and dystroglycan (α- and β-DG) complexes (Kanagawa and Toda 2006). In normal skeletal muscle, dystrophin mediates the attachment of the cytoskeleton of the muscle fibers to the endomysial basement membrane, the muscle connective tissue composed by ECM proteins such as laminin-2, perlecan and collagen IV. The direct interaction with the ECM, primarily with laminin-2, is mediated by members of the DG complex, where the extracellularly localized α-DG anchors the sarcolemma to the basement membrane through interaction with β-DG. This protein associates with dystrophin, which is anchored to the F-actin filaments of the cytoskeleton, thus creating the mechanical link. SG proteins also play a crucial role in stabilizing these interactions, mainly through SG-DG interaction. Therefore, the loss of SG proteins leads to sarcoglycanopathies (Blain and Straub 2011), other forms of autosomal recessive MDs, such as Limb-girdle MD, which cause disruption of the DGC. Skeletal muscles lacking dystrophin are more fragile and weaker than healthy ones, and they undergo contraction-induced skeletal fiber death (necrosis), which is the main pathophysiological DMD mechanism.

## Muscle regeneration in muscular dystrophies

In normal skeletal muscle repair, quiescent muscle stem cells residing between the basement membrane and the sarcolemma, formally called satellite cells (SC; Pax7^+^), are activated to regenerate and repair the damaged tissue. These stem cells proliferate upon activation, and some daughter cells are further committed into a myogenic differentiation program primarily controlled by transcription factors of the Muscle regulatory factors (MRF) family such as MyoD, myogenin and others. The newly formed myocytes fuse with preexisting damaged myotubes to facilitate growth and regeneration, with central nuclei as their histological hallmark (Bentzinger et al. 2013).

SCs are typically defined as stem cells that give rise to skeletal muscle cells. However, it is widely accepted that they have the potential to acquire different fates. For instance, SCs can achieve osteogenic, adipogenic, and fibro-mesenchymal differentiation depending on extrinsic signals such as soluble factors and the presence of immune cells (Pessina et al. 2015; Madaro et al. 2019). Surprisingly, dystrophin can also regulate SC differentiation. Essentially, activated stem cells undergo asymmetric division to give rise to one self-renewal pool that maintains the stem cell population and another committed pool that further differentiates. Dystrophin is expressed in SCs and regulates polarity-dependent asymmetric divisions. The absence of dystrophin in SCs impairs these mechanisms, leading to a reduced number of asymmetric divisions and committed cell number, resulting in a reduced number of myogenin^+^ cells (Dumont et al. 2015).

In inherited MDs, the newly differentiated muscle cells lack components of the DGC that are later participants of the characteristic continuous degenerative/regenerative cycle. This phenomenon leads to exhaustion in the regenerative capacity of SC (Jejurikar and Kuzon 2003; Ribeiro et al. 2019), which, with the development of fibrosis, produces the muscle's degeneration.

Although fibrosis is detrimental for parenchyma function in all tissues, the controlled-acute deposition of ECM coupled with an inflammatory response is critical for proper skeletal muscle repair. However, dystrophic muscles illustrate the opposite side of the dysregulated process.

## Inflammation in muscular dystrophies

Inflammation is a necessary response for the removal of damaged tissue and pathogens. Since skeletal muscle from MDs is continuously prone to degeneration, there is a persistent inflammatory response leading by immune cells and resident skeletal muscle cells (Tidball et al. 2018). Inherited MDs have no cure and glucocorticoids are nowadays the standard accepted treatment because they improve muscle strength (Shieh 2018). However, these drugs can have several adverse effects. Evidence of inflammation in DMD comes from pharmacological research using prednisolone-immunosuppressed *mdx* mice, which found reduced infiltrating inflammatory cell numbers and cell adhesion molecules in *mdx* muscles (Wehling-Henricks et al. 2004; Angelini 2007), showing that inflammation is an essential process in MDs.

Inflammation can be acute or chronic depending on its duration, types of cells implicated, and the response’s resolution. Due to the constant regeneration/degeneration cycles in MDs, the response is chronic and accompanied by periodic activation of acute inflammation (Tidball et al. 2018). The main cell types that infiltrate *mdx* skeletal muscles are macrophages (MP) and lymphocytes, typical cells in chronic inflammation (McDouall et al. 1990; Porter et al. 2002). However, there are other immune cells that can participate in the pathology, such as neutrophils and eosinophils.

### Relevancy of macrophages in acute and dystrophic inflammation

MPs are the most prominent inflammatory cells in *mdx* mice (Tidball et al. 2018; Wehling-Henricks et al. 2004). These cells are derived from circulating monocytes that differentiate into MPs when reaching the target tissue. These cells can be subclassified into two different phenotypes; classically activated M1-MP and alternatively activated M2-MP, corresponding to pro-inflammatory and anti-inflammatory MPs, respectively (Gordon 2003). In response to acute injury, the first cells to infiltrate the muscle are mostly leukocytes belonging to the myeloid lineage, mainly neutrophils and monocytes/MP. Pro-inflammatory monocytes (Ly6C^high^) are recruited to the injured muscle and differentiate towards a pro-phagocytic inflammatory MP (M1 phenotype) to clean the damaged tissue by removing cellular debris. These cells then shift towards an anti-inflammatory phenotype (M2 phenotype) required for proper tissue regeneration (Arnold et al. 2007). However, in chronic injury, like in MDs, inflammatory cells persist in the tissue modifying the well-orchestrated microenvironment for muscle regeneration.

As mentioned before, M1-MPs, positive for CD68, are present in the initial/acute inflammatory response. They can be activated by inflammatory signals such as lipopolysaccharides from bacteria, and interferon-γ (IFN-γ) secreted from Th1 T cells and others. M1-MPs express inducible nitric oxide synthetase (iNOS). This enzyme uses arginine to produce citrulline and nitric oxide, triggering muscle fiber damage through nitric oxide-dependent cytotoxicity. As expected, iNOS null *mdx* mice show reduction in muscle fiber damage compared with *mdx* mice (Villalta et al. 2009). On the other hand, M2-MPs, positive for CD206 and CD163, and activated by IL-10 and IL-4, are implicated in myoblast proliferation, and anti-inflammatory and fibrotic responses (Wynn and Vannella 2016; Villalta et al. 2011a). M2-MPs secrete IL-10 and TGF-β (Hsieh et al. 2018). They also express arginase I, which uses arginine, the same substrate of iNOS, to produce proline, which is required for collagen synthesis (Wynn 2004). The metabolism of arginine in M2-MP promotes cardiac and muscle fibrosis in *mdx* mice (Wehling-Henricks et al. 2010), suggesting that the M1-MP is an essential cell type that mediates the acute inflammatory response, whereas the M2-MP phenotype contributes to fibrosis development.

It is hypothesized that skeletal muscle cells can also promote M1-MP activation by signaling toll-like receptors (TLR) and cytokines' release. TLR are membrane or cytosolic pattern recognition receptors activated by different molecules such as damage-associated molecular patterns including the high mobility group box 1 protein (HMGB1), a nuclear-resident protein released in response to cell damage. HMGB1 binds TLR4 on the membrane surface and activates a signaling pathway that promotes the release of pro-inflammatory cytokines (Klune et al. 2008). Skeletal muscle from *mdx* mice expresses a wide range of TLR, including TLR4 (Henriques-Pons et al. 2014). HMGB1 is increased in *mdx* mice muscles, and TLR4-deficient *mdx* mice show reduced inflammation and skeletal muscle dystrophy, probably due to a shift of the MP phenotype towards an anti-inflammatory M2-biased MP (Giordano et al. 2015). The canonical adaptor Myeloid differentiation primary response 88 (MyD88) transduces the signal of the HMBG1/TLR pathway. In accordance with the role of TLR4, *mdx* mice show increased MyD88 expression. Moreover, satellite cell-specific deletion of MyD88 in *mdx* mice disrupts myogenesis, associated with the aggravation of the fibrotic phenotype and increased mRNA expression of CD206 and CD163, markers of M2 MP (Gallot et al. 2018).

As discussed above, different signals from distinct cells can regulate the MP phenotype and eventually contribute to muscle regeneration defects. However, increased abundance of MPs in *mdx* is still necessary. Madaro et al. showed that macrophage depletion worsens the dystrophic phenotype of *mdx* mice, increasing fibrosis and adipogenesis (Madaro et al. 2019). Mechanistically, MP depletion leads to a change in SC fate, developing a novel phenotype with adipogenic potential. These cells can form adipocytes in a dystrophic MP-depleted muscle context, reinforcing that SC fate can be regulated by its niche where MPs play a pivotal role (Madaro et al. 2019). However, MP depletion also increases the population of FAPs and neutrophils, which can regulate fibrosis and myogenesis (Arecco et al. 2016; Uezumi et al. 2014a).

### Granulocytes degenerate the tissue in muscular dystrophies

Neutrophils are constituents of the granulocyte family of leukocytes that also includes basophils and eosinophils. Neutrophils are sensitive inflammatory cells that express myeloperoxidase (MPO), a cytolytic enzyme responsible for muscle cells damage in the early stages of DMD (Nguyen et al. 2005). Moreover, these cells can also interrupt skeletal muscle regeneration by disrupting myoblast myogenesis through the production of elastase, a serine protease augmented in *mdx* mice muscles (Arecco et al. 2016).

Eosinophils, another cell type that belongs to the granulocyte family, are incremented in muscles of *mdx* mice and DMD patients (Cai et al. 2000; Wehling-Henricks et al. 2008). There are strong indications that these cells can promote fibrosis. During acute injury, eosinophils (Siglec F^+^ CD11b^+^), which are an important source of IL-4 essential for muscle regeneration, are recruited to the muscle. IL-4 promotes the proliferation of FAPs. Interestingly, eosinophil-depleted mice show decrease proliferation of FAPs after muscle injury. A similar outcome is observed in IL-4/IL-13 null mice (Heredia et al. 2013). Whether IL-4-rich eosinophils are important participants in the development of fibrosis in chronic injury by increasing the rate of FAPs is still unknown. Moreover, IL-4 is a potent inducer of MP's M2 phenotype (Villalta et al. 2009). Therefore, eosinophil-secreted IL-4 could be an unexpected signal for MP polarization. Supporting this role albeit probably through a different mechanism, muscles from *mdx* mice that do not express the major basic protein 1 (MBP-1), a constituent of the cytotoxic granules of eosinophils, show lower hydroxyproline concentrations and weaker immunostaining for collagen I, III, and IV in muscle sections from heart, limbs, and diaphragm (Wehling-Henricks et al. 2008).

T lymphocytes are acquired immunity-specialized cells that mediate cellular immunity. The number of T cells is increased in DMD muscle biopsies with a predominance of CD4^+^ over CD8^+^ T cells (McDouall et al. 1990). These cells are critical in MDs since antibody-dependent depletion of both CD4^+^ and CD8^+^ T cells improves muscle histopathology in *mdx* mice (Spencer et al. 2001). In agreement with the mechanism of CD8^+^-mediated cell lysis, *mdx* mice lacking perforin, a membrane pore-forming protein, show fewer apoptotic myonuclei and MP invaded-muscle fibers than *mdx* mice (Spencer et al. 1997). Moreover, immunodeficient *mdx* mice that lack the thymus (an essential organ for T cell maturation) have less collagen content in skeletal muscles compared with *mdx* mice (Morrison et al. 2000), confirming critical participation of both T cells subpopulations in MDs. Recently, regulatory T cells (T regs; Foxp3^+^), a specialized CD4^+^ lymphocyte, have been described as critical regulators of muscle regeneration. These cells accumulate in the muscle upon acute or chronic injury (*mdx* mice) and controls MPs phenotype dynamics through the regulation of cytokine production (discussed below in the cytokine section) (Villalta et al. 2014; Panduro et al. 2018). Importantly, depletion of T regs during acute injury increases inflammation and fibrosis. Moreover, T regs depletion in *mdx* worsens inflammation and myofiber damage, suggesting that these cells can be a valuable immune cell population for therapeutic approaches.

The infiltration of the skeletal muscle by several immune cells due to damage is a well-orchestrated process. The correct influx and efflux of myeloid and lymphoid cells into the muscle after an injury is essential for the proper establishment of a pro-regenerative microenvironment in the tissue. Nevertheless, persistent inflammation due to chronic maintenance of immune cells with pro-fibrotic, pro-myonecrotic, or anti-regenerative capabilities is very deleterious to the muscle architecture. Pharmacological and genetic modification of immune cells revealed their critical participation in MDs and in the establishment of fibrosis. Thus, efforts to find molecules with immune modulatory properties are required. Nevertheless, extracellular inflammatory molecules (cytokines and chemokines) add another spatio-temporal regulation of inflammation in the skeletal muscle and are significantly involved in MDs.

### Cytokines in muscular dystrophies

*Interleukin-1β* is the main pro-inflammatory cytokine involved in the acute response acting locally or systemically. IL-1β is increased in the serum on *mdx* mice (Mancio et al. 2017) and colocalizes with MPs in the diaphragm (Hnia et al. 2008), suggesting that this cell is an important source of this cytokine in MDs. In relation to myogenesis, IL-1β-stimulated myogenic cells isolated from non-dystrophic mice showed delayed differentiation. However, IL-1β was able to completely suppress myogenesis in myogenic cells isolated from dystrophic mice through the induction of Jagged1 expression, a Notch signaling pathway ligand (Nagata et al. 2017). This result suggests that a pro-inflammatory context commanded by IL-1β could be a critical factor in the loss of regenerative capacity of DMD muscles. It has been suggested that IL-1β can also indirectly regulate the fate of FAPs through MPs. The adipogenic differentiation of FAPs could be reduced by factors released by IL-1β-polarized MPs (Moratal et al. 2018).

*The tumor necrosis factor* family of cytokines are mediators of acute inflammation, acting on EC and neutrophils promoting its activation. In many other cell types, TNF induces apoptosis through TNF receptor 1 (TNFR1) death domain signaling. The most studied member of the TNF family, TNF-α, is extensively involved in skeletal muscle inflammation, and is expressed in myocytes, EC and leukocytes, among other cell types (Collins and Grounds 2001; Peterson et al. 1985). MP-secreted TNF-α is greatly relevant to control fibrosis establishment due to prevent FAPs accumulation by inducing its apoptosis (Lemos et al. 2015). Skeletal muscles from DMD patients have increased number of TNF-α-expressing myofibers that correlates with regenerating fibers (Tews and Goebel 1996; Kuru et al. 2003), supporting a role for this cytokine on muscle fiber inflammation. Moreover, the expression of TNF-α mRNA also increases in other muscle fibrosis models such as denervation and barium chloride-induced chronic damage (Contreras et al. 2016). Consistently, the pharmacological inhibition of TNF-α binding to its receptor in *mdx* mice decreases the percentage of necrotic muscle fiber (Hodgetts et al. 2006), suggesting TNF-α as a key myonecrotic factor.

*Interleukin-6* is another pro-inflammatory cytokine released by the phagocytic system (MPs, dendritic cells) and other cells whose function overlaps with TNFα and IL-1β in inflammatory responses. In healthy skeletal muscle, IL-6 mRNA is barely detectable, whereas upon injury it is overexpressed, especially in myoblasts (Kurek et al. 1996). This high expression declines to similar levels as those found in the uninjured control muscles after one week. Therefore, IL-6 is implicated in the inflammatory response during skeletal muscle regeneration. Indeed, IL-6 functions as a pro-myogenic factor that regulates the proliferation of SCs and myoblasts (Okazaki et al. 1996; Serrano et al. 2008; Al-Shanti et al. 2008). The expression levels of muscle IL-6 are increased in DMD patients and correlate positively with age (Messina et al. 2011), suggesting a chronic role for this cytokine associated with the degenerative stage. Contrary to DMD patients, increased IL-6 levels in *mdx* mice decay around 24 weeks of age. IL-6 overexpression in the *mdx* mice exacerbates the dystrophic phenotype by increasing necrotic and regenerative fibers, as well as increasing the expression of inflammatory mediators such as TNFα and NF-κB associated with impaired muscle function (Pelosi et al. 2015). These effects are consistent with IL-6 sustaining the continuous cycle of degeneration/regeneration that governs MD.

Contrary to the classical view as a pro-inflammatory cytokine, IL-6 function in MDs is controversial, and it has also been postulated as an anti-inflammatory cytokine. Treatment of *mdx* mice with a monoclonal antibody against the IL-6 receptor, which blocks locally and systematically IL-6 signaling, enhances the number of infiltrated mononuclear cells and increases the expression of the intracellular adhesion molecule 1 (ICAM-1) in the gastrocnemius muscle. ICAM-1 expressed by EC is essential for leukocyte transmigration (Kostek et al. 2012). Nevertheless, the blockage of the IL-6 receptor does not improve muscle function and regeneration, probably due to the lack of IL-6-dependent cellular regulatory mechanisms, such as myogenesis, during regeneration (Okazaki et al. 1996; Serrano et al. 2008; Al-Shanti et al. 2008). In DMD, the depletion of SCs and the increased number of FAPs is the typical scenario. Overexpression of IL-6 in *mdx* mice causes increased activation of SCs, but of at the same time reduces the pool of SCs and increases the number of FAPs (Pelosi et al. 2015).

*Interleukin-10* is a homodimer that exerts one of the most crucial anti-inflammatory activities. IL-10 is mainly released by Th2 cells and M2-MPs and promotes the deactivation of M1-MP, inhibiting the secretion of some pro-inflammatory cytokines such as TNFα and IL-12 (Howes et al. 2014). Accordingly, IL-10 induces activation of M2-MPs, while IL-10 mutant mice experience reduced regeneration capacity associated with increased number of necrotic fibers (Deng et al. 2012), confirming the importance of IL-10 in promoting skeletal muscle regeneration by regulating the MP subtype population. This mechanism is supported by previous reports showing that monocytes/MP recruited to muscles upon injury, which express M2-MPs-like cytokine profiles (IL-10 and TGFB1) and stimulated with M2-MPs-inducers cytokines (IL4 and IL-10), can promote SC differentiation (Arnold et al. 2007). In the context of MDs, IL-10 transcript levels in *mdx* mice differ among different muscles. At four weeks of age IL-10 mRNA is overexpressed in hamstring muscles, while later on (12 weeks) it is overexpressed in quadriceps muscle (Villalta et al. 2009, 2011a). MPs isolated from *mdx* muscles respond to IL-10, increasing Arginase I expression and decreasing iNOS expression, and reducing myotube damage in co-culture assays. Notably, IL-10 deficient *mdx* mice show exacerbated muscle fiber damage and muscle function impairment associated with a reduced M2-MPs population compared to *mdx* mice (Villalta et al. 2011a). Thus, IL-10 expression and signaling are critical for driving skeletal muscle dystrophy progression.

*Interferons* are a group of cytokines classified into two types. Type I interferons (IFN-α secreted by MP and IFN-β secreted by fibroblasts) regulate cellular responses associated with the anti-viral state and class I major histocompatibility complex (MHCI). On the other hand, type II IFN-γ, released from Th1 T cells, is implicated in adaptive immunity through the activation of M1-MP. This cytokine is induced in a time-dependent manner during acute skeletal muscle injury, reaching its highest levels around 5 days post-injury. Its highest levels correlate with increased abundance of MPs, T cells, natural killer cells, and myoblasts within the muscle since it is expressed by all these cell types (Cheng et al. 2008). Interestingly, Foxp3^+^CD4^+^ regulatory T cells (Treg) are critical regulators of IFN-γ/MP dynamics. Injured muscle in mice depleted of Tregs presents elevated numbers of IFN-γ-expressing cells and enhanced IFN-γ response by MPs, associated with a more pro-inflammatory phenotype (Panduro et al. 2018). Therefore, Treg is a novel immune cell that regulates MP phenotype and muscle physiology through IFN-γ production.

IFN-γ plays a pivotal role in regulating skeletal muscle regeneration and fibrosis. Strategies using blocking antibodies against the IFN-receptor in skeletal muscle and the IFN-γ null mice showed that the skeletal muscle shows decreased regeneration, and the presence of collagen deposits following cardiotoxin-induced damage. The skeletal muscle also exhibits fewer MPs, iNOS expression, and myoblast differentiation (Cheng et al. 2008).

How is IFN-γ associated with fibrosis? IFN-γ-mediated activation of the IFN-receptor triggers a signaling pathway that involves the Janus kinase 1/Signal transducer that regulates gene expression. The mechanism by which blocking IFN-γ expression/signaling alters muscle regeneration and collagen deposition could be attributable to the capacity of IFN-γ to act as an antagonist of TGF-β1 signaling by inhibiting the formation of the TGF-β receptor-Smad3 complex (Ulloa et al. 1999). IFN-γ inhibits muscle-derived fibroblast growth and TGF-β1 effects after muscle injury. Interestingly, in TGF-β1-overexpressing myoblasts that acquire a myofibroblast-like phenotype, IFN-γ inhibits the expression of α-SMA and vimentin (Foster et al. 2003). In laceration-induced muscle injury, there is increased collagen deposition that can be attenuated by the administration of soluble IFN-γ, which also improves muscle regeneration and function (Foster et al. 2003).

Regarding MD, *mdx* mice show increased IFN-γ mRNA levels compared to wild type mice, and IFN-γ stimulation of MPs from *mdx* mice shows enhanced iNOS expression (Villalta et al. 2009). Moreover, 12-week-old IFN-γ null *mdx* mice, show less muscle fiber damage and improved function compared to *mdx* mice of the same age. This is probably due to reduced number of MI-MP and increased number of M2-MPs, which would facilitate myogenesis (Villalta et al. 2011b). These effects were not seen at the 4-weeks-stage, showing that the expression of IFN-γ is detrimental in late, but not in early MD stages.

### Chemokines in muscular dystrophies

Other soluble factors that mediate inflammation responses are chemokines. These low molecular weight proteins are cytokines with chemoattractant properties, which recruit different population of cells to the tissues where are synthesized and secreted. Chemokines are classified based on the position of cysteine residues in their amino-terminal end in C, C–C, C–X–C, and C–X–3C families (Baggiolini 2001). Several chemokines and chemokine receptors are transiently overexpressed after acute skeletal muscle injury, such as monocyte chemoattractant protein 1 (MCP-1) or C–C motif chemokine ligand 2 (CCL2), macrophage inflammatory protein (MIP)-1α, MIP-1β, and some G protein-coupled receptors such as C–C motif chemokine receptor 2 (CCR2) and CCR5 (Warren et al. 2005).

The relevance of CCR2, the receptor for CCL2, CLL7, and CCL8, has been extensively studied. CCR2 mRNA and protein expression are transiently upregulated in skeletal muscle after injury. The CCR2 protein co-localizes with MP and myogenic precursor cell markers (Warren et al. 2004, 2005). Consistent with this localization, C2C12 myoblasts express CCR2 and respond to its ligand CCL2, which induces cell proliferation through a CCR2/G_αi_/ERK1/2-dependent mechanism (Yahiaoui et al. 2008). Moreover, CCL2 administration into cardiotoxin-injured tibialis anterior muscle in mice transiently decreases myogenin expression, a transcription factor that governs myoblast differentiation, suggesting that CCL2 can also regulate myoblast behavior. CCR2 null mice show decreased myofiber size and increased fibroblast markers, fat and collagen. Increased muscle fat deposition after cardiotoxin injury was also observed in mice lacking CCL2 expression (Martinez et al. 2010). These tissue characteristics are associated with impaired clearance of macrophages and neutrophils that persist in the muscle 14 days after injury (Warren et al. 2005; Martinez et al. 2010). Functionally, these muscles also show lower strength recovery rates 14 days after injury (Warren et al. 2004), suggesting that CCR2 expression and signaling is an essential regulator of skeletal muscle repair.

MDs display a persistent abundance of inflammatory cells. Gene expression analysis showed the overexpression of distinct chemokines and chemokine receptors in *mdx* mice, including CCL2, CCL6, CLL7, CCL8, CCL9, CCR1, CCR2, and CCR5 (Porter et al. 2002, 2003). CXCL-14, which belongs to the C–X–C chemokine family, and its receptor CXCR4 are also upregulated in *mdx* mice and are specifically involved in lymphocyte recruitment. However, CCL7 and CCL8 are not overexpressed at the protein level (Porter et al. 2003).

Recently, increased transcript levels of CCL2, CCL7, CCL8, CCL12, and CCR2 were found in the diaphragm and tibialis anterior muscles from *mdx* mice (Mojumdar et al. 2014). This study also showed that the MP population is attenuated in the muscle of 6-week-old CCR2-deficient *mdx* mice but not in 12-week-old *mdx* mice, suggesting that more than one mechanism regulates MP recruitment and persistence during late stages of the pathology. Moreover, there are differences between the phenotypes of early and late MP populations. Indeed, at 6 weeks of age, the increment in the M1-MPs population within the muscle of *mdx* mice is reversed in CCR2-deficient *mdx* mice to levels like those present in WT mice. This was also associated with normalization in the expression of CD206 by MPs in CCR2-deficient *mdx* mice, which was decreased in *mdx* mice (Mojumdar et al. 2014). Whether this mechanism also regulates the MP population in the regenerative stage (12-week-old mice) is still unknown.

Histopathological analysis showed that myonecrosis and fibrosis (measured as hydroxyproline content) improved to WT levels in CCR2-deficient *mdx* mice compared to *mdx* mice. In addition, the administration of an antibody targeting CCR2 (fusokine) to *mdx* mice causes a decrease in the number of macrophages and in collagen abundance. Fusokine also increases fiber size like what is seen if CCR2-deficient *mdx* mice (Mojumdar et al. 2014). These data show the critical role of CCR2 expression and signaling in dystrophic muscle and strongly suggest that the recruitment of MPs and phenotype dynamics are its main mechanisms.

In conclusion, cytokines and chemokines play pivotal roles in regulating immune cell functions associated with muscular dystrophies. Cytokines can also act on resident muscle cells regulating their pro-regenerative functions. Several cytokines and chemokines are modulators of fibrosis and together with immune cells are essential participants of skeletal muscle diseases.

## Fibrosis development in muscular dystrophies

The nature of inflammation, commanded by the type of damage, affects how the skeletal muscle architecture is recovered. The establishment of chronic inflammation determine a distinct microenvironment within the tissue modifying cell–cell communications and signaling pathways primarily controlled in acute inflammation. Effects such as constantly cytokine and chemokine’s release, impaired immune cells persistency, ECM-producing cells propagation, and myogenic cells differentiation defects often conclude in tissue degeneration and fibrosis, a typical result of chronic diseases like DMD.

Fibrosis is characterized by the accumulation of ECM components such as collagen and fibronectin due to the imbalance between synthesis and degradation, which generates a scar-like phenotype and progressive muscle strength loss.

The cell type mainly responsible for the synthesis and deposition of ECM in skeletal muscle are myofibroblasts. They are α-SMA positive and are characterized by the presence of contractile microfilament bundles in the cytoplasm (stress fibers) that give them contractile properties (Sandbo and Dulin 2011). The number of myofibroblasts is increased in muscles from DMD patients and in the *mdx* mice (DMD mouse model) (Contreras et al. 2016; Hori et al. 2011). These cells derive mainly from fibro/adipogenic progenitors (FAPs), PDGFRα positive cells, that are also increased in muscle from DMD patients and in fibrotic models such as muscle denervation (Contreras et al. 2016, 2019; Uezumi et al. 2014b).

### Major signaling pathways that regulate fibrosis

The biomolecular mechanisms underlying fibrosis include the participation of many factors and pathways. The crosstalk among them at different levels has been described, adding new evidence for a deleterious vicious cycle that promote an uncontrolled fibrogenic program.

*Renin-Angiotensin System (RAS)*, signals through its classical and non-classical axes. The classical axis, which includes Angiotensin II and its type-1 receptor (AT-1), seems to have a pro-fibrotic effect since it induces fibronectin and collagen-III expression in the muscle cell line C2C12 (Cabello-Verrugio et al. 2011a). The expression of these ECM proteins has also been observed in skeletal muscle from mice treated with Angiotensin II (Morales et al. 2014), while pharmacological inhibition of the Angiotensin II synthesizing enzyme (angiotensin-converting enzyme, ACE) and AT-1 by enalapril and losartan respectively, reduces ECM accumulation and improves muscle strength in *mdx* mice (Morales et al. 2013a; Cohn et al. 2007). On the other hand, RAS's non-classical axis, composed of Angiotensin 1–7 and its receptor Mas, seems to play a protective role in the muscle of *mdx* mouse since treatment with Angiotensin 1–7 triggers a decrease in fibronectin and collagen levels and increases skeletal muscle strength (Acuña et al. 2014). These opposite effects between the classical and non-classical RAS pathways have been observed in other biological processes such as the regulation of vascular tone. Therefore, their opposite effects on fibrosis is not unexpected.

*Transforming growth factor type β* (TGF-β) is another relevant molecule in fibrosis. It is involved in regulating many cellular processes such as differentiation, apoptosis, and proliferation under physiological conditions and also in fibrotic tissue (Massague 2012; Biernacka et al. 2011; Kim et al. 2018). In DMD and *mdx* muscles, TGF-β is upregulated (Bernasconi et al. 1999; Ishitobi et al. 2000) and contributes to ECM deposition mainly through its canonical pathway, which involves phosphorylation of SMAD proteins (Ismaeel et al. 2019). The pro-fibrotic effect of TGF-β in *mdx* and muscle regenerating animals is prevented by the use of a monoclonal antibody against TGF-β (Cohn et al. 2007; Andreetta et al. 2006) and proteoglycans such as decorin and biglycan (Kolb et al. 2001; Casar et al. 2004). Moreover, increased expression of ECM proteins, such as type I collagen, has been observed in wild type tibialis anterior muscle injected with TGF-β1 (Li et al. 2004). These results confirm the extraordinary ability of TGF-β to induce ECM even in a non-fibrotic context. This pro-fibrotic effect of TGF-β can be explained at least partially by this factor's ability to promote the differentiation of FAPs into myofibroblast-like cells in vitro (Uezumi et al. 2010).

TGF-β promotes the expression of the matricellular protein Cellular communication network factor 2/connective tissue growth factor (CCN2/CTGF) in different cell types (Chen et al. 2000; Cheng et al. 2015). Interestingly, the effect of TGF-β on CCN2 induction is enhanced in myotubes in vitro and in vivo in skeletal muscle fibers by the HIF1α-mediated hypoxia signaling pathway (Valle-Tenney et al. 2020). These observations are essential for two reasons: (1) hypoxia has been related to fibrosis in different organs (Darby and Hewitson 2016) and (2) CCN2 is a remarkable ECM remodeling factor and is augmented in DMD.

CCN2 expression is increased in *mdx* mice and in denervated muscles (Morales et al. 2013b, 2018; Rebolledo et al. 2019). The experimental overexpression of CCN2 in wild-type muscle using a sequence-containing adenovirus causes increased expression of collagens, fibronectin, and α-SMA, suggesting an expansion of the myofibroblast population. Furthermore, the return of CCN2 to basal levels reverses the CCN2 triggered phenotype (Morales et al. 2011). Moreover, reduced expression of CCN2 in the *mdx* mouse triggers decreased expression of fibrotic markers and the formation of necrotic regenerative foci (Morales et al. 2013b, 2018). Moreover, treatment with a neutralizing monoclonal antibody against CCN2 (FG3019 or Pamrevlumab) produces a similar effect in fibrotic markers (Morales et al. 2013b). In agreement with these results, blocking CCN2 with Pamrevlumab can reverse the fibrosis triggered by overused-induced muscle injury in rats (Barbe et al. 2020). Taken together, these data strongly suggest that CCN2 is involved in DMD's pathophysiology and supports the ongoing clinical trial that is testing the use of anti-CCN2 antibodies in DMD patients (NCT02606136, NCT04371666).

Summarizing, the RAS, TGF-β and CCN2 pathways are involved in the onset, maintenance, and progression of muscular fibrosis. Discovering tools that modulate these pathways can open treatment possibilities for chronic diseases such as DMD.

## The ATX/LPA/LPARs axis

Lysophosphatidic acid (LPA, 1-or 2-acyl-sn-glycerol 3-phosphate) is a small (430–480 Da) cytokine-like, membrane-derived, bioactive glycerophospholipid composed of a glycerol backbone, a single saturated or unsaturated fatty acid chain, and a phosphate group.

LPA has a half-life of approximately 3 min in circulation (Tomsig et al. 2009), depending on its synthesis and degradation rates. LPA is produced in the intra- and extracellular environments. Intracellular synthesis is mediated by phospholipases A1 and A2 using cell membrane phosphatidic acid as substrate (Aoki et al. 2008). It can also be generated by acylation of glycerol 3-phosphate, a reaction catalyzed by glycerophophate acyltransferase, and by phosphorylation of monoacylglycerol through a monoacylglycerol kinase (Pages et al. 2001). Extracellular LPA is synthesized mainly from lysophosphatidylcholine and lysophosphatidylserine by ATX, a lysophospholipase D secreted enzyme, encoded by the *Enpp2* gene. ATX is the main source of circulating LPA since heterozygotes *Enpp2* mutant mice have a 50% reduction in LPA plasma levels compared to wild-type mice (D'Souza et al. 2018). LPA has been found in several tissues and biological fluids (Yung et al. 2014), but adipose tissue has been proposed as the primary source of LPA in circulation (Ferry et al. 2003; Dusaulcy et al. 2011).

As a regulatory mechanism, LPA accumulation decreases ATX synthesis (Benesch et al. 2015). LPA can be degraded to monoacylglycerol by lipid-phosphate phosphatases (LPP 1–3). Therefore, LPP1-deficient mice shown high plasma LPA levels (Tomsig et al. 2009).

**Fig. 1:**
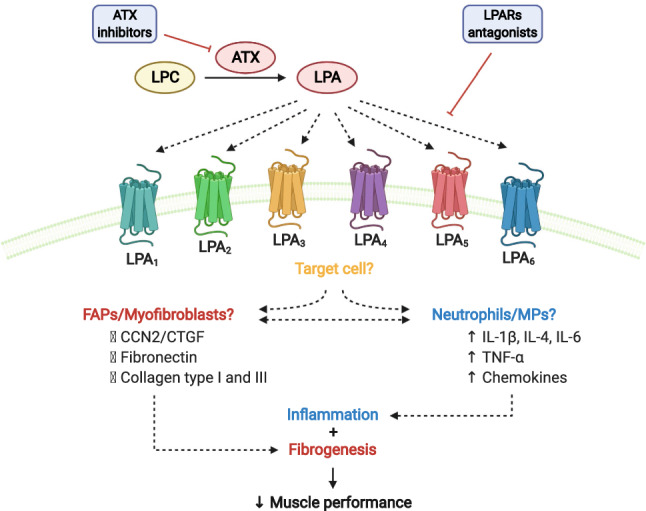
Model for the role of ATX/LPA/LPARs axis in MDs. ATX synthesizes LPA from lysophosphatidylcholine (LPC). LPA activates G-protein-coupled receptors (LPA_1–6_), triggering various cellular signaling in different cell types. LPA increases the expression of pro-fibrotic factors such as CCN2, and ECM proteins such as fibronectin, type I and III collagen in FAPs and myofibroblast. In neutrophils and MPs, LPA signaling increases inflammatory cytokines such as IL-1β, IL-4, IL-6, TNF-α, and some chemokines. This condition may lead to the activation of inflammation and fibrotic responses, impairing muscle performance. Created with biorender.com

LPA signals through six different G-protein coupled receptors (GPCRs), called LPA_1_ through LPA_6_. All of them are rhodopsin-like GPCRs with seven transmembrane helices. LPA_1_ through LPA_3_, belong to the family of endothelial differentiation genes (Edg), while the other more structurally distant receptors, LPA_4_ through LPA_6_, are associated with the purinergic family of receptors (Yung et al. 2014). LPA regulates many cellular processes through its receptors, triggering multiple downstream molecular pathways, such as Rho/Rock, PI3K/Akt and PLC/IP3 to mediate cytoskeleton remodeling, cell survival, intracellular calcium transients, differentiation, and other cellular processes under physiological and pathophysiological conditions (Hemmings and Brindley 2020; Valdes-Rives and Gonzalez-Arenas 2017). The axis is summarized in Fig. 1.

The relative expression of the LPA receptors varies in different tissues and organs, depending on their role. Although in skeletal muscle the expression and distribution of LPA receptors are not fully known, there is some evidence that supports their presence in this tissue, being LPA_1_ the most highly expressed (Anliker and Chun 2004; Jean-Baptiste et al. 2005; Zderic and Hamilton 2012). However, the roles that LPA and its receptors are playing in the skeletal muscle under physiological and pathological conditions are still unknown. Despite that, LPA signaling has been well characterized in inflammation and fibrosis, and is an possible new attractive participant in muscle biology.

## The LPA/LPARS/ATX axis in the inflammatory response

An increasing amount of evidence places LPA as a potent modulator of inflammation in different tissues such as lung, liver, and cancer-related-organs (reviewed in Valdes-Rives and Gonzalez-Arenas 2017; Gonzalez-Arenas et al. 2008). In the lungs, LPA induces neutrophil chemoattractant activity in epithelial cells, increasing neutrophil influx. Mechanistically, in response to LPA bronchial epithelial cells increase the expression of the IL-8 chemokine (neutrophil-specific C-X-C chemokine) through activity regulation of NF-κB and AP-1, classical transcription factors involved in cytokine regulation (Cummings et al. 2004; Saatian et al. 2006). This mechanism also requires G protein isoforms G_i_ and G_12/13,_ and protein kinase C (PKC) isoform PKCδ. Inhibition of these transducers attenuates LPA-induced IL-8 expression and NF-κB activation (Cummings et al. 2004). However, more than one signaling pathway can converge in order to regulate cytokine expression. In this context, (Saatian et al. 2006) LPA could also regulate IL-8 secretion through two independent mechanisms involving p38/NF-κB and JNK/AP-1, where LPA_1_ and LPA_3_ are also required. This evidence supports the regulation of cytokine production by LPA through different molecular pathways (Fig. 1).

Non-resident immune cells are recruited from the bloodstream to the inflamed tissue through EC adhesion and communication. Using intravital microscopy, Kranig et al. (Kranig et al. 2019) showed that in *mdx* mice, there is augmented leukocyte extravasation and leukocyte expression of LFA-1 and Mac-1. These molecules are members of the β2-integrin family that interact with ICAM-1 in EC, a process necessary for leukocyte transmigration. Consistent with its role in inflammation, it is possible that LPA could regulate leukocyte recruitment since EC from human umbilical veins (HUVECs) increase ICAM-1 expression after LPA treatment. This response requires LPA_1_ expression, which promotes EC-monocyte adhesion (Lee et al. 2004; Lin et al. 2007). *mdx* mice also show increased expression of cell adhesion molecules mRNAs such as endothelial VCAM-1 (Porter et al. 2002). Further studies on the modulation of adhesion molecules in skeletal muscles as a response to LPA are necessary.

As in bronchial epithelial cells, EC also responds to LPA, increasing the expression and secretion of IL-8 and MCP-1 through an LPA_1_-, Gi-, Rho-, and NF-κB-dependent mechanism. Furthermore, LPA also induces the expression of IL-1β in these cells, mediated by LPA3. Notably, the induction of IL-8 and MCP-1 are dependent on the activation of IL-1R (Lin et al. 2006, 2007). Of note, the conditioned medium from these EC cultures has chemoattractant activity, consistent with these chemokines' functions. Accordingly, the silencing of LPA_1_ and LPA_3_ inhibits LPA-induced chemoattractant activity. However, LPA_1_ but not LPA_3_ silencing impairs LPA-enhanced EC-monocyte adhesion, consistent with IL-8/MCP-1 and ICAM-1 expression pattern. These results show that LPA could be an essential element in promoting an inflammatory response by increasing chemokine and adhesion molecule expression and subsequent leukocyte recruitment.

LPA-regulated MCP-1 expression is not exclusive of EC. Human smooth muscle cells also increase MCP-1 expression in response to LPA in a Rac1- and ROS generation-dependent fashion (Kaneyuki et al. 2007). Moreover, IL-6, another critical cytokine involved in MDs is also regulated by LPA in smooth muscle cells. LPA increases IL-6 expression at the messenger and protein levels. This regulation also requires the LPA_1_ receptor and a signaling pathway that involves the activation of the G_i/o_ protein isoform, PKC, and p38 (Hao et al. 2010).

Regarding the skeletal muscle, MCP-1 expression increases in LPA-treated C2C12 myoblast. LPA is also involved in increasing myoblast proliferation (Tsukahara and Haniu 2012). These data suggest that LPA can mediate leukocyte recruitment by regulating chemokine secretion in different resident cells of the skeletal muscle, such as EC and smooth muscle cells, including the vasculature and myoblasts.

Monocyte differentiation and MP activation can be regulated by different signals (Shi and Pamer 2011). LPA is an inducer of CD11b^+^ monocyte differentiation towards F4/80^+^ MP. This mechanism depends on the Akt/mTOR signaling pathway and PPARγ as the downstream regulator (Ray and Rai 2017). Importantly, LPA can also promote human monocyte differentiation to macrophages by the same mechanism. It is not known if LPA regulates MP subtype specification. Nevertheless, there is some evidence suggesting MP phenotype modulation by LPA. LPA has been shown to induce the expression of IL-1β from MP by a mechanism that depends on G_i_/Rho signaling and the generation of ROS (Chang et al. 2008), which is consistent with a pro-inflammatory phenotype. On the other hand, LPA increases the number of CD86^+^ and CD206^+^ microglia cells (the macrophages of the brain's immune system), referred to as pro-and anti-inflammatory markers respectively (Plastira et al. 2020). Thus, expression analysis of specific subtype markers in MP would help determine their role in the skeletal muscle in response to LPA and its potential participation in inflammation and fibrosis.

There is only one report that analyzes the role of LPA administration on inflammation of chronic injured skeletal muscle. Induced chronic injury of the rotator cuff muscles promotes an increment of the macrophage and neutrophil population associated with the onset of fibrosis and fat deposition. As expected, intraperitoneal administration of LPA enhances MP and neutrophil infiltration and worsens the fibrotic and adipose phenotype seen in damaged-muscle (Davies et al. 2017).

The modulation of the ATX/LPA/LPARs axis also regulates the inflammatory response in different organs. In a mouse model for Chron's disease, a chronic inflammatory bowel pathology, the symptoms can be attenuated with the administration of PF8380 (a pharmacological inhibitor of ATX). The expression of IL-4 (here postulated as a pro-fibrotic cytokine), IL-13, and TNF-α, as well as leucocyte infiltration in the ileal tissue, are increased in a Chron's disease-mice model and can be significantly attenuated in mice treated with PF-8380 (He et al. 2018), decreasing the cellular differentiation defects.

In a myocardial infarction model, injured mice show increased LPA levels that correlate positively with the number of inflammatory cells in plasma (Tripathi et al. 2020). The injured heart also showed increased expression and activity of ATX. The administration of PF8380 just after injury attenuated the augmented infiltration of neutrophils, pro-inflammatory monocytes, and MP. This inhibitor also decreased mRNA expression of several inflammatory cytokines and chemokines compared to vehicle-treated mice. As expected, the size of scarred tissue, measured through collagen staining, was also decreased in PF8380-treated mice after infarction challenge, improving the tissue's functional recovery (Tripathi et al. 2020). Also, the global or adipocyte-specific deletion of the ATX gene can attenuate the increased levels of inflammatory cytokines in adipose tissue and lipid accumulation in the liver in obesity associated with a high-fat diet (Brandon et al. 2019).

Finally, in the brain, administration of LPA can modulate the inflammatory context. In a mouse model of acute brain inflammation in mice (LPS administration), there were increased LPA levels and augmented expression of some LPARs in the brain. Furthermore, isolated microglia, showed increased expression of cytokines, such as IL-1b, IL-6, and TNF-a, as well as chemokines CCL5, CXCL2, and CXCL10 when treated with LPA (Plastira et al. 2020).

## The ATX/LPA/LPARs axis in the development of fibrosis

Numerous studies propose a pro-fibrotic role for the ATX/LPA/LPARs axis in different organs and diseases. LPA is increased in bronchoalveolar lavage fluid (BAL) from the bleomycin-induced lung fibrosis model. In agreement with these results, LPA is increased in BAL and in exhaled breath condensate from patients with idiopathic pulmonary fibrosis (Tager et al. 2008; Montesi et al. 2014). LPA and ATX are also upregulated in the liver from patients with HCV infection, which is accompanied by liver fibrosis in advanced stages of the disease (Watanabe et al. 2007). Similarly, there are increased levels of extracellular LPA in explants from fibrotic kidneys induced by a unilateral ureteral obstruction (UUO) (Pradere et al. 2007). These data strongly suggest that there is local LPA secretion and signaling in the fibrotic organ. Even though there are no reports of LPA levels in muscles from patients with skeletal muscle fibrosis-driving diseases, intraperitoneal LPA injections worsen atrophy and fibrosis induced by tendon nerve injury in rotator cuff muscle (Davies et al. 2017).

Studies that modulate the function or the presence/absence of LPA receptors yielded the most promising results that suggest a role for the ATX/LPA/LPARs axis in fibrosis pathogenesis (Fig. 1). There is increasing data suggesting that pharmacological blockage or genetic deletion of LPA receptors could prevent the development of induced fibrosis in different organs. The primary LPA receptors that appeared to be involved in fibrosis are LPA_1_ and LPA_3_. LPA1 transcripts are increased in the kidney after UUO, and LPA_1_ KO mice show decreased fibrosis markers such as collagen III. The LPA_1_ and LPA_3_ inhibitor Ki16425 triggered a similar response (Pradere et al. 2007).

In the same way, lung fibrosis induced by radiation shows dramatic increases in LPA_1_ and LPA_3_ transcripts. Treatment with the LPA_1_/LPA_3_ inhibitor VPC12249, reduces collagen deposition and prevents the induction of pro-fibrotic cytokines such as CCN2 and TGF-β in the lung (Gan et al. 2011). The positive effects of inhibiting LPA receptors in lung fibrosis were also replicated in the bleomycin induced model (Ohashi and Yamamoto 2015). In the murine model SOD1-G93A that mimics amyotrophic lateral sclerosis (ALS), which causes skeletal muscle fibrosis, the treatment with AM095, an LPA1 inhibitor, causes motor skills improvement evaluated by grip strength, rotarod, and runtime (Gento-Caro et al. 2021). These results support the use of inhibitors of the ATX/LPA/LPARs axis in the treatment of fibrotic disorders, as addressed in numerous clinical trials, mainly in idiopathic pulmonary fibrosis and scleroderma. It would be interesting to know if those inhibitors can be useful tools in treating muscle fibrosis in diseases such as DMD.

The differentiation of resident cells towards a myofibroblast-like phenotype could be one of the roles of LPA in the development/maintenance of fibrosis (Fig. 1). A mouse peritoneal fibrosis model shows that the accumulation of αSMA positive cells is prevented in the absence of LPA_1_ (Sakai et al. 2013). In skeletal muscle, one of the primary sources of myofibroblasts are FAPs, which are increased under fibrotic conditions such as those found in *mdx* and in denervated muscles (Contreras et al. 2016). It is necessary to investigate if LPA is involved in the differentiation of FAPs towards a myofibroblast phenotype in muscles from these animals. Furthermore, it is possible that LPA could be mediating pro-fibrotic effects by inducing other biological behaviors in FAPs, such as migration and proliferation, as has been shown in different cell types in cancer (Stähle et al. 2003; Yamada et al. 2008).

**Fig. 2:**
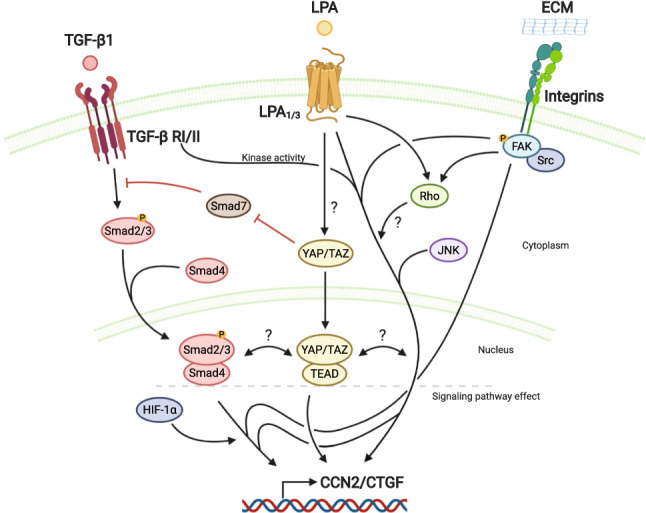
The interplay between CCN2/CTGF regulatory signaling pathways. Different signaling pathways of varying nature can converge to regulate the pro-fibrotic factor CCN2. For instance, the serine/threonine kinase receptor activatied by TGF-β signaling promotes Smad2/3-dependent induction of CCN2, a response synergistically potentiated by low oxygen availability. Such receptor activity is also required for LPA_1/3_-dependent LPA induction of CCN2. Adding more complexity, LPA-dependent signaling also requires JNK activation to induce CCN2, while Rho GTPase could be involved as well, being activated by LPA and the integrin/FAK axis. Furthermore, this axis also supports the effects of TGF-β and LPA. Hippo/YAP/TAZ signaling pathway emerges as the central sensor of those responses because it can be modulated by different extracellular cues and it can induce CCN2. This pathway can feedforward into TGF-β/Smad signaling by reducing Smad7 levels. Importantly, the YAP/TAZ pathway is activated by GPCRs such as those that are part of the LPA/LPARs axis. Whether LPA and TGF-B1, coupled with the integrin/FAK axis, converge with YAP/TAZ to regulate CCN2 is unknown, but would add a new spatial component for the control their pro-fibrogenic activity. Created with biorender.com

It seems that LPA is closely related to the TGF-β pathway since, in Tenon's fibroblasts, the contraction induced by TGF-β is inhibited by Ki16425, which also downregulates the SMAD 2/3 proteins (Wen et al. 2019). Similarly, Ki16425 prevents the migration and proliferation of these cells. It has been proposed that the synergistic fibrotic effect of LPA and TGF-β could also be explained by the participation of the Hippo/YAP/TAZ pathway (Zmajkovicova et al. 2020). This pathway includes several proteins, with the Yes-asocciated protein (YAP) and the transcriptional co-activator with PDZ-binding motif (TAZ) being the key final effectors. These proteins require translocation to the nucleus to exert their function as co-transcriptional activators. The Hippo signaling pathway is mediated by G-protein-coupled receptor (GPCR) ligands such as LPA (Cai and Xu 2013). Besides, the YAP/TAZ pathway is required for TGF-β/ Smad signaling since decreased YAP/TAZ levels induces Smad 7, a known negative regulator of the TGF- β pathway (Qin et al. 2018) (Fig. 2). In the same line, our laboratory has demonstrated that C2C12 myoblasts respond to LPA, increasing the expression of CCN2 through a mechanism that requires the activation of the TGF-β receptor I (Cabello-Verrugio et al. 2011b). Preliminary experiments using the same cell line have shown that inhibition of LPA receptors 1 and 3 (using Ki16425) prevents the induction of CCN2 by TGF-β, suggesting that there is crosstalk between both pathways. Accordingly, YAP binding to the CCN2 promoter results in its induction (Zhao et al. 2008). It would be interesting to determine if the induction of CCN2 by LPA and TGF- β is prevented by inhibiting the YAP/TAZ pathway using tools such as the inhibitor Verteporfin or siRNAs targeting components of this signaling pathway in myoblasts (Fig. 2). In a recent publication, Riquelme-Guzmán showed that LPA and TGF-β require integrin signaling for the induction of CCN2 in myoblasts, adding the ECM/integrin axis as a new factor in LPA signaling and supporting the idea of cross-talk between LPA and TGF-β pathways in muscle cells (Riquelme-Guzman et al. 2018). This functional interaction among LPA/TGF-β/integrin has also been suggested by Xu et al. based on results that demonstrated that LPA induces αvβ6-mediated TGF-β activation in epithelial cells through RhoA and Rho kinase (Xu et al. 2019).

Taken together, these data strongly suggest that the ATX/LPA/LPARs axis could be part of an unexplored pro-fibrotic program in skeletal muscle pathologies causing muscular fibrosis such as DMD and Limb-girdle MD.

## The ATX/LPA/LPARS axis as a novel therapeutic target in muscular dystrophies

According to the evidence summarized in this review, many investigations show promising effects of pharmacological inhibitors of the ATX/LPA/LPARs axis in murine models of kidney, skin, and lung fibrosis (Gan et al. 2011; Ohashi and Yamamoto 2015; Pradere et al. 2008; Castelino et al. 2011; Ninou et al. 2018). Moreover, the inhibition of ATX in a murine chronic colitis model improved inflammation, downregulating IL-6, and STAT3 in the colon (Dong et al. 2019).

There is a recent report on the structure-based finding of new inhibitors of ATX, which seeks to improve their specificity and optimize possible treatments (Magkrioti et al. 2020). Until now, clinical trials testing the potential use of ATX and LPA_1_ axis inhibitors in humans have been conducted predominantly on idiopathic pulmonary fibrosis and systemic sclerosis (NCT03798366, NCT01766817, NCT04308681).

The ATX/LPA/LPARs axis is emerging as an attractive target for the development of new treatments for inflammatory and fibrotic diseases, including muscular dystrophies. However, there are still no studies linking LPA with these pathologies. It is necessary to study the effect of ATX/LPA/LPARs inhibitors in murine models of MDs.

## Concluding remarks

MDs are a wide range of skeletal muscle degenerative diseases with no cure or satisfactory treatment. DMD is the most lethal type, with a life expectancy of 30 years in affected patients. Skeletal muscle structure and function are impaired due to low regeneration capability, chronic inflammation, and fibrosis.

Skeletal muscle inflammation is a necessary response for normal regeneration after damage. However, when this response becomes chronic, characterized by the constant maintenance of immune cells and the overexpression of pro-fibrotic factors, it triggers fibrosis. The functional skeletal muscle tissue is replaced by disorganized fibrotic and adipogenic stromal tissue, which is deleterious for muscle performance.

Several lines of evidence demonstrated that inflammation plays a pivotal role in the induction of fibrosis. For instance, the imbalance and dysregulation of immune cell populations and the overexpression of a wide range of cytokines and chemokines are associated with fibrosis. Moreover, inflammatory cells and cytokines with anti-muscle regeneration properties are also overrepresented in dystrophic muscles. Therefore, inflammation can govern two major scenarios related to MDs.

**Fig. 3:**
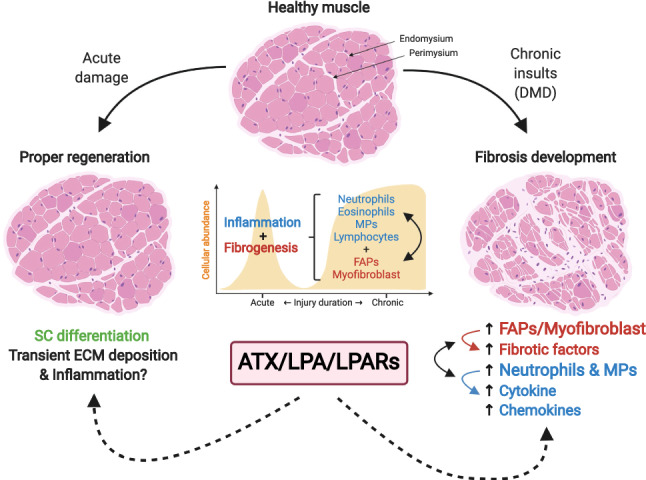
Proposed functions for the ATX/LPA/LPARs axis in acute and chronic damage. Normal skeletal muscle regeneration is affected by the nature of the damage. Acute injuries are resolved by a well-orchestrated and transient increase of inflammatory cells and ECM-producing cells. The crosstalk between each cell population establishes a scaffolding network for proper muscle regeneration. On the other hand, as occurs in DMD patients, chronic insults result in persistent accumulation of both cell populations with enhanced capacities. We propose that the expression of ATX, and as a consequence an increase in LPA-mediated signaling, could be necessary for muscle regeneration by regulating SCs differentiation. Whether this axis is essential for acute ECM deposition and inflammation in skeletal muscle is unknown. Activation of LPA-mediated signaling induces some pathophysiological responses present in different organs and tissues in DMD. Some of them correspond to the induction of cytokines, chemokines, and fibrotic factors expression and the accumulation of myofibroblast, MPs, and neutrophils. If these effects are present in the skeletal muscle, this axis would be an attractive signaling pathway in future therapeutic considerations for MDs. Created with biorender.com

For several years, research was focused on improving muscle function through the targeting of molecules with pro-inflammatory or pro-fibrotic functions. The finding of novel molecules with the capacity to control both inflammation and the fibrotic response would help develop new potential therapeutic drugs to combat MDs. Here, we propose the novel ATX/ LPA/LPARs axis as a new target. This axis can modulate the expression of cytokines and chemokines and the abundance of immune cells in tissues affected by chronic inflammation. It can influence the establishment of fibrosis, regulating the overexpression of pro-fibrotic factors in other organs, such as lung, liver, and kidney. Figure 3 summarizes the axis's components in MDs and the potential effect of the pathway on different target cells, resulting in increased inflammatory and fibrotic responses that affect muscle performance.

Many responses regulated by this axis are present in dystrophic muscles. However, there is little evidence of its function in this tissue. Despite this, there are already significant contributions (Tsukahara and Haniu 2012; Davies et al. 2017; Cabello-Verrugio et al. 2011b; Riquelme-Guzman et al. 2018). Recently, Sah et al. demonstrated that ATX expression and function are required for the myogenic differentiation of C2C12 myoblasts. Furthermore, using injured muscles from mice, they found that ATX mRNA expression increases substantially on day four after injury, and that in vivo deletion of the ATX-encoding gene (Enpp2) negatively affects the regeneration process (Sah et al. 2020).

**Fig. 4:**
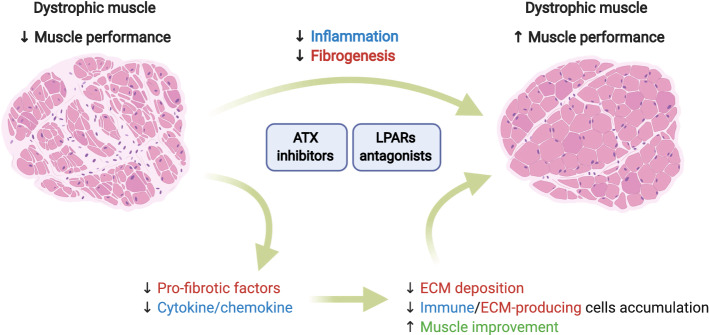
The ATX/LPA/LPARs axis as a potential therapeutic target for MDs. Muscle degeneration due to inflammation and fibrosis is the most influencing issue on muscle performance in MDs. The regulation of inflammation and fibrogenesis is the most attractive way to fight MDs. LPA induces pro-fibrotic factors, cytokines, and chemokines. We propose that the use of LPARs antagonists or inhibitors of ATX activity could attenuate the generation of these molecules, decreasing ECM protein deposition and the continued presence of immune and ECM-producing cells, therefore improving muscle regeneration and performance. Created with biorender.com

In conclusion, the ATX/LPA/LPARs axis is a pathway with promising roles in the pathophysiology of MDs. Figure 4 shows the ATX/LPA/LPARs axis as a potential therapeutic target in MDs. Its involvement in the modulation of inflammation and fibrosis makes it an attractive pharmacological target for several chronic diseases. Future research focusing on its role in the skeletal muscle may open new possibilities for understanding muscle homeostasis and disease.

## Abbreviations

ATX: Autotaxin
CTGF or CCN2: Connective tissue growth factor
DMD: Duchenne muscular dystrophy
DGC: Dystrophin-associated glycoprotein complex
EC: Endothelial cells
ECM: Extracellular matrix
FAPs: Fibro/adipogenic progenitors
iNOS: Inducible nitric oxide synthetase
IFN: Interferon
IL: Interleukin
ICAM-1: Intracellular adhesion molecule 1
LPA: Lysophosphatidic acid
LPAR: Lysophosphatidic acid receptor
MP: Macrophages
MDs: Muscular dystrophies
RAS: Renin-angiotensin system
SC: Satellite cells
TGF-β: Transforming growth factor
TNF: Tumor necrosis factor
